# Comprehensive transcriptome analysis identifies novel molecular subtypes and subtype-specific RNAs of triple-negative breast cancer

**DOI:** 10.1186/s13058-016-0690-8

**Published:** 2016-03-15

**Authors:** Yi-Rong Liu, Yi-Zhou Jiang, Xiao-En Xu, Ke-Da Yu, Xi Jin, Xin Hu, Wen-Jia Zuo, Shuang Hao, Jiong Wu, Guang-Yu Liu, Gen-Hong Di, Da-Qiang Li, Xiang-Huo He, Wei-Guo Hu, Zhi-Ming Shao

**Affiliations:** Department of Breast Surgery, Fudan University Shanghai Cancer Center, 270 Dong-An Road, Shanghai, 200032 P.R. China; Cancer Institute, Fudan University Shanghai Cancer Center, 270 Dong-An Road, Shanghai, 200032 P.R. China; Department of Oncology, Shanghai Medical College, Fudan University, 270 Dong-An Road, Shanghai, 200032 P.R. China; Institutes of Biomedical Sciences, Fudan University, Shanghai, P.R. China

**Keywords:** Molecular subtypes, Messenger RNA, Long non-coding RNA, Triple-negative breast cancer, Transcriptome analysis

## Abstract

**Background:**

Triple-negative breast cancer (TNBC) is a highly heterogeneous group of cancers, and molecular subtyping is necessary to better identify molecular-based therapies. While some classifiers have been established, no one has integrated the expression profiles of long noncoding RNAs (lncRNAs) into such subtyping criterions. Considering the emerging important role of lncRNAs in cellular processes, a novel classification integrating transcriptome profiles of both messenger RNA (mRNA) and lncRNA would help us better understand the heterogeneity of TNBC.

**Methods:**

Using human transcriptome microarrays, we analyzed the transcriptome profiles of 165 TNBC samples. We used k-means clustering and empirical cumulative distribution function to determine optimal number of TNBC subtypes. Gene Ontology (GO) and pathway analyses were applied to determine the main function of the subtype-specific genes and pathways. We conducted co-expression network analyses to identify interactions between mRNAs and lncRNAs.

**Results:**

All of the 165 TNBC tumors were classified into four distinct clusters, including an immunomodulatory subtype (IM), a luminal androgen receptor subtype (LAR), a mesenchymal-like subtype (MES) and a basal-like and immune suppressed (BLIS) subtype. The IM subtype had high expressions of immune cell signaling and cytokine signaling genes. The LAR subtype was characterized by androgen receptor signaling. The MES subtype was enriched with growth factor signaling pathways. The BLIS subtype was characterized by down-regulation of immune response genes, activation of cell cycle, and DNA repair. Patients in this subtype experienced worse recurrence-free survival than others (log rank test, *P* = 0.045). Subtype-specific lncRNAs were identified, and their possible biological functions were predicted using co-expression network analyses.

**Conclusions:**

We developed a novel TNBC classification system integrating the expression profiles of both mRNAs and lncRNAs and determined subtype-specific lncRNAs that are potential biomarkers and targets. If further validated in a larger population, our novel classification system could facilitate patient counseling and individualize treatment of TNBC.

**Electronic supplementary material:**

The online version of this article (doi:10.1186/s13058-016-0690-8) contains supplementary material, which is available to authorized users.

## Background

Contrary to their description in previous studies as being useless transcripts, long noncoding RNAs (lncRNAs) are emerging as important regulators in gene regulation and other cellular processes [1–6]. Recent studies have proved that lncRNAs are tightly correlated with disease processes, including cancer [4, 7–10]. The roles of lncRNAs in breast cancer have also been widely researched, and a number of novel mechanisms have been proposed [1–3, 11]. As with other cancers, lncRNAs are involved in several developmental and tumorigenic processes of breast cancer. Liu et al. [2] reported that the lncRNA *NIKLA*, can directly interact with the functional domains of signaling proteins, serving as a class of NF-κB modulators to suppress breast cancer metastasis. Another lncRNA, *BRCA4*, was reported to direct cooperative epigenetic regulation downstream of chemokine signals, and its expression correlated with advanced breast cancer [12]. Gupta et al. [13] observed increased expression of the lncRNA, *HOTAIR*. in primary breast tumors and metastases, and *HOTAIR* expression level in primary tumors was a powerful predictor of eventual metastasis and death. Considering the important role of lncRNAs in breast cancer tumorigenesis and development, the study of lncRNAs might aid in understanding the nature of this malignant disease.

One of the most aggressive breast cancer subtypes is triple-negative breast cancer (TNBC), which lacks estrogen receptor (ER) and progesterone receptor (PR) expression and human epidermal growth factor receptor 2 (HER2) amplification [14, 15]. TNBC represents approximately 10–20 % of all breast cancers and has a larger tumor size, higher grade, more positive lymph nodes, and poorer prognosis than other subtypes of breast cancer [16, 17]. Due to the heterogeneity of the disease and the absence of well-defined molecular targets, treatment of TNBC remains a clinical challenge. Differences were observed in the responses of patients with TNBC to the same adjuvant chemotherapy. Thus, further classifying this aggressive disease subtype and treating patients accordingly is a top priority and would greatly benefit patients.

Several former studies have achieved significant progresses in classifying TNBC. By analyzing publically available expression data for messenger RNA (mRNA), Lehmann et al. [18] advanced the knowledge of TNBC and classified TNBC into six subtypes: 1) luminal androgen receptor positive (LAR); 2) claudin-low-enriched mesenchymal (M); 3) mesenchymal stem-like (MSL); 4) immune response (M); and two cell cycle-disrupted basal subtypes, 5) basal-like-1 (BL1) and 6) basal-like-2 (BL2). In the present study, we refer to this as the Lehmann/Pietenpol classification. However, a subsequent study using the Lehmann/Pietenpol classification could not readily distinguish BL1 and BL2 tumors [19]. Moreover, with recent developments in high-throughput (gene sequencing) technology, our knowledge of breast cancer is ever expanding, and a classification based merely on gene expression levels may be insufficient (for prospective individualized cancer treatment). A new classification system based on the integrated expression profiles of mRNAs and lncRNAs might offer more comprehensive data and identify stable subtypes and subtype-specific targets.

Collectively, we questioned the possibility and utility of subtyping TNBCs using whole-transcriptome expression analysis. By integrating the expression profiles of both mRNAs and lncRNAs, we successfully classified 165 TNBC tumors into four distinct subtypes, each displaying unique gene expression and ontology. Furthermore, we identified subtype-specific lncRNAs and predicted their possible biological functions using co-expression network analysis. Our novel classification system could facilitate individualized treatment for patients with TNBC if validated in other reliable cohorts.

## Methods

### Patient recruitment

The present prospective observational study was initiated on 1 January 2011. Patients who were diagnosed with malignant breast cancer and willing to participate in the study were recruited. A total of 165 consecutive patients treated in the Department of Breast Surgery at Fudan University Shanghai Cancer Center (FUSCC) from 1 January 2011 to 31 December 2012 were enrolled according to the following inclusion criteria: 1) female patients diagnosed with unilateral invasive ductal carcinoma with phenotype ER–, PR–, and HER2–; 2) pathologic examination of tumor specimens performed by the Department of Pathology at FUSCC. The ER, PR and HER2 status was reconfirmed by two experienced pathologists (WTY and RHS) based on immunochemical analysis and *in situ* hybridization [20]; 3) patients without any evidence of metastasis at diagnosis; and 4) sufficient frozen tissue for further research. Patients with breast carcinoma *in situ* (with or without microinvasion) and inflammatory breast cancer were excluded. Clinicopathological characteristics (including age, menopausal status, tumor histologic type, tumor size, lymph node status, histologic grade, ER, PR, HER2, Ki67, and adjuvant therapies) and local and distant extent of disease (evaluated by chest computed tomography (CT), bone scan, abdominal ultrasound, bilateral mammography, breast ultrasound or magnetic resonance imaging (MRI)) were collected [21].

Follow up of patients in the cohort was completed on 31 December 2014. The median length of follow up was 13.9 months (interquartile range, 8.6–21.1 months). Our definition of recurrence-free survival (RFS) events included: the first recurrence of invasive disease at a local, regional, or distant site; the diagnosis of contralateral breast cancer; and death from any causes. Patients without events were censored at the last follow up.

All tissue samples included in this study were obtained with approval of the independent ethical committee/institutional review board at Fudan University Shanghai Cancer Center Ethical Committee, and each patient signed an informed consent form.

### Sample preparation and microarray experiment

Tumor tissues were macro-dissected to avoid the influence of stromal tissues (<10 %). The percentage of tumor cells was confirmed to be 90 % or more in all breast cancer specimens. Total RNA was isolated from 165 frozen TNBC samples using the Rneasy Plus Mini Kit (Qiagen, Valencia, CA, USA). The purity and quantity of total RNA were estimated by measuring absorbance at 260 nm (A260) and 280 nm (A280) with RNase-free water as a blank control, using a NanoDrop 2000 spectrophotometer (Thermo Scientific, Wilmington, DE, USA). Only when the ratio of A260/A280 was within 1.9–2.1, were the extracted RNAs deemed as pure and suitable for future experimentation. Microarray analysis was performed using the Affymetrix Human Transcriptome Array 2.0 (HTA 2.0) GeneChips (Affymetrix, Santa Clara, CA, USA) as previously described [22].

### The mRNA-lncRNA-based TNBC subtyping and the Lehmann/Pietenpol classification

We performed *k*-means clustering and consensus clustering to determine the optimal number of stable TNBC subtypes. Cluster robustness was assessed by consensus clustering using agglomerative *k*-means clustering (1,000 iterations), with average linkage on the 165 TNBC profiles using the 2,535 most differentially expressed genes (SD >0.65) (Gene Pattern version 3.2.1, http://www.broadinstitute.org/cancer/software/genepattern/). The optimal number of clusters was determined from the empirical cumulative distribution function (CDF), which plots the corresponding empirical cumulative distribution, defined over the range, and from calculation of the proportion increase in the area under the CDF curve [18]. In addition, we considered the number of patients in each subtype. If there were fewer than five patients in one subtype, we deemed the classification as unstable. Thus, the optimum number of clusters moved to the minor number.

The Lehmann/Pietenpol classification system was established by analyzing 587 TNBC gene expression profiles from 21 publicly available datasets [18]. The authors have developed a web-based subtyping tool for classifying TNBC samples based on their collected gene expression meta-data [23]. Using this web-based algorithm [23] (http://cbc.mc.vanderbilt.edu/tnbc/), we obtained subtypes of our samples in the Lehmann/Pietenpol classification system. Spearman’s correlation analysis was used to assess the relationship between the Lehmann/Pietenpol classification system and our novel system.

### Gene Ontology (GO) and pathway analysis

GO analysis was applied to analyze the main function of the subtype-specific genes according to the GO database, which is the key functional classification of the National Center for Biotechnology Information (NCBI). The analysis can organize genes into hierarchical categories and uncover the gene regulatory network based on biological process and molecular function [24–26]. Meanwhile, pathway analysis was used to determine the significant pathways of the differential genes according to the Kyoto Encyclopedia of Genes and Genomes database (KEGG) [27]. The Pearson chi-square test and Fisher’s exact test were used to select the significant pathway.

### Co-expression network analysis

To identify interactions between mRNAs and lncRNAs, we constructed co-expression networks [28]. We pre-processed the data using the median expression value of all transcripts and then screened for differentially expressed lncRNAs and mRNAs among subtypes. For each pair of genes analyzed, we calculated the Pearson correlation and chose pairs (only lncRNA-mRNA) with significant correlation in order to construct the network (*P* <0.05). To make a visual representation, only those with the strongest correlation (correlation coefficient ≥0.95) were included in the renderings. The co-expression networks were drawn using Cytoscape 2.8.2 [29], which is open-source software for integration, analysis and visualization of biological networks.

### Statistical analysis

All analyses were performed according to the reporting recommendations for tumor marker prognostic studies (REMARK) for prognostic and tumor marker studies, and the respective guidelines of microarray-based studies for clinical outcomes. Frequency tabulation and summary statistics were used to characterize the data distribution. Student’s *t* test was utilized to compare continuous variables, and the Pearson chi-square test was employed for the comparison of categorical variables. Survival curves were constructed using the Kaplan–Meier method and compared between subtypes with the log rank test. Survival analyses were performed using SPSS 20.0 (SPSS Inc., Chicago, IL, USA). All tests were two-sided, and *P* <0.05 was regarded as significant, unless otherwise stated.

### Microarray data

Microarray data have been deposited into the Gene Expression Omnibus (GEO) database (http://www.ncbi.nlm.nih.gov/geo/) [GEO:GSE76250].

## Results

### Transcriptome profiling of TNBC reveals four stable molecular subtypes

**Fig. 1:**
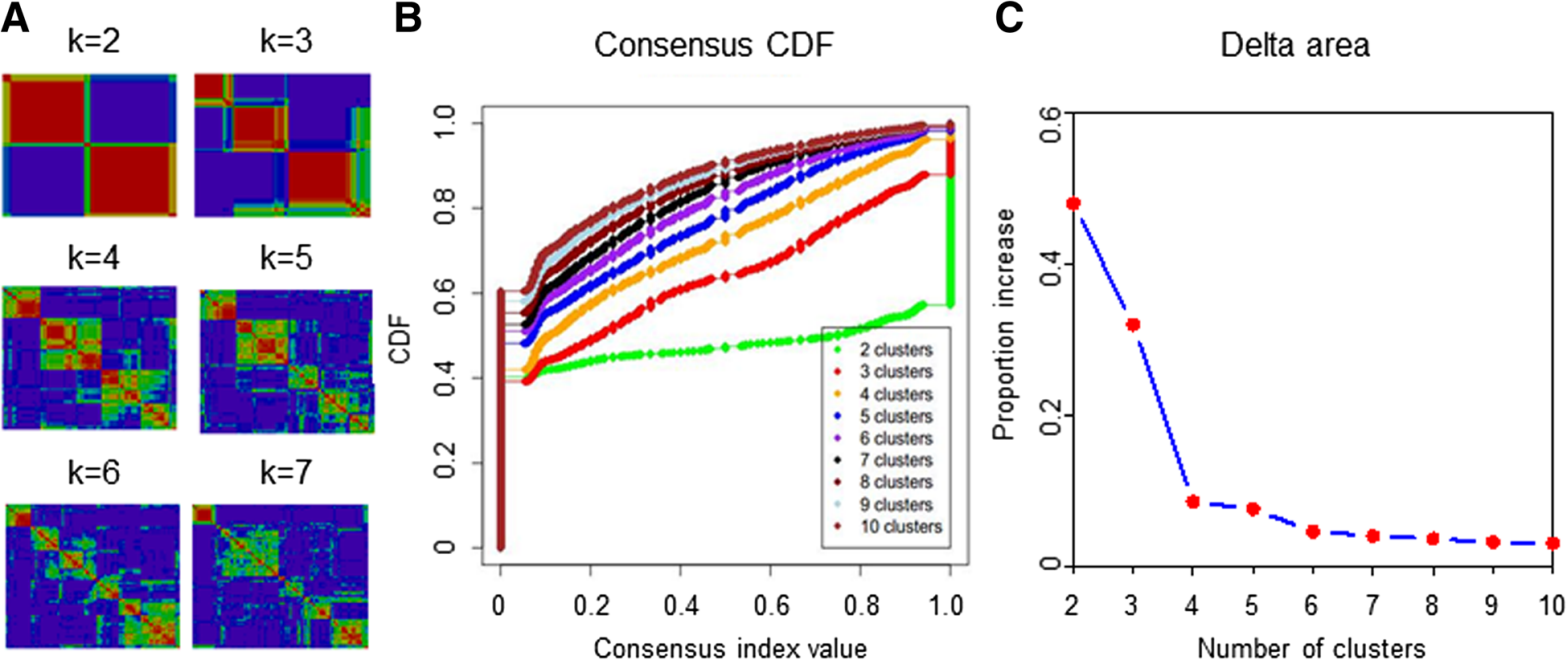
The identification of novel subtypes of triple-negative breast cancer. **a** Consensus clustering displaying the robustness of classification. **b** Consensus empirical cumulative distribution function (*CDF*) of all given cluster numbers. **c** Plot of delta area changes with number of clusters

**Fig. 2:**
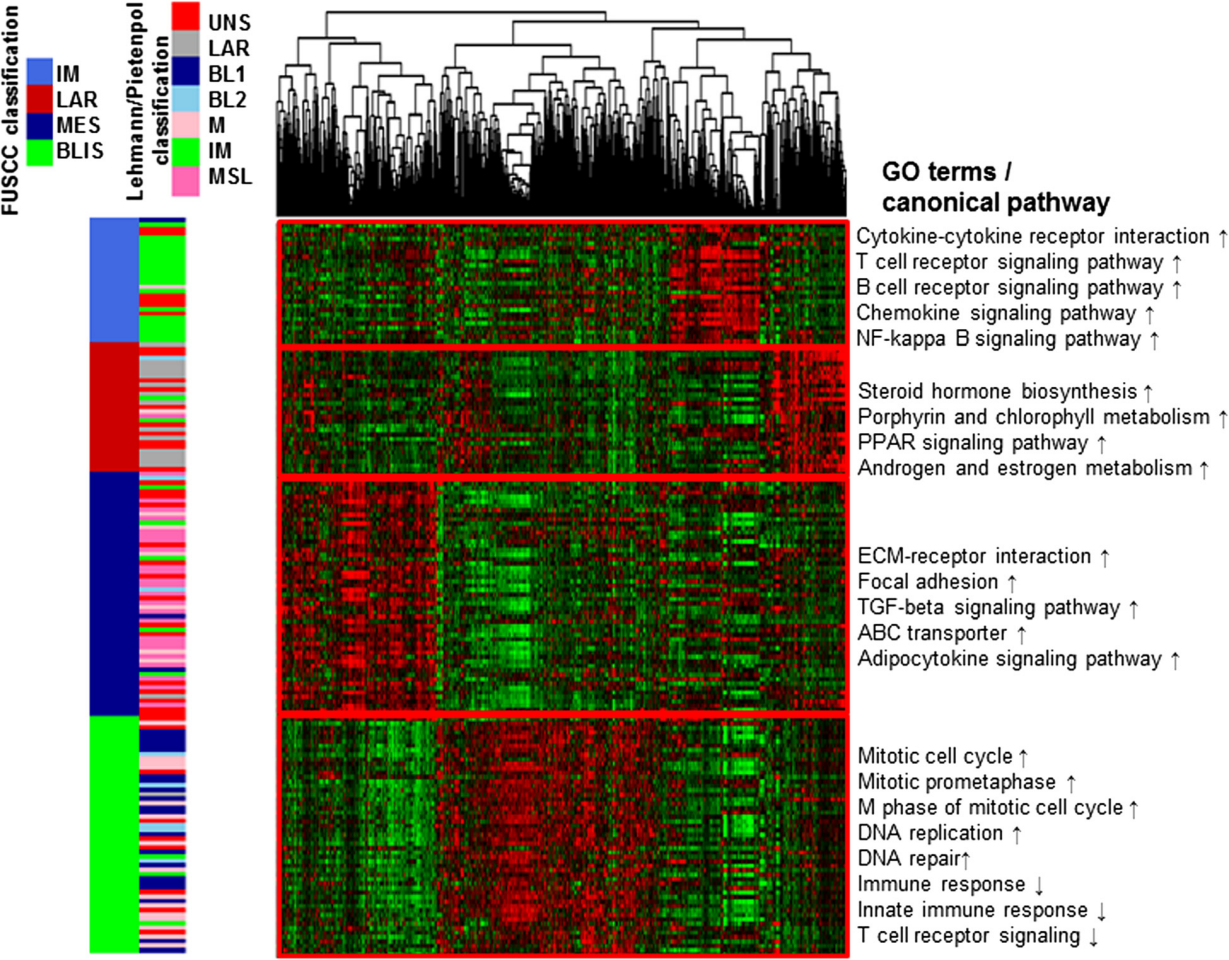
A heat map shows the relative expression of the top differentially expressed RNAs (SD >0.65) in each subtype. Top Gene Ontology (*GO*) and canonical pathways of each subtype are shown (*left*). *Upward-pointing arrow* upregulated function, *downward-pointing arrow* downregulated function. *FUSCC* Fudan University Shanghai Cancer Center, *IM* immunomodulatory, *LAR* luminal androgen receptor, *MES* mesenchymal-like, *BLIS* basal-like and immune suppressed, *BL* basal-like, *M* claudin-low-enriched mesenchymal, *MSL* mesenchymal stem-like, *ECM* extracellular matrix, *TGF* transforming growth factor

According to the inclusion criteria, a total of 165 TNBC samples qualified for the present study. To identify global differences in transcriptome profiles in TNBC subtypes, we performed *k*-means clustering on the most differentially expressed mRNAs and lncRNAs (SD >0.65). All 165 TNBC tumors were classified into four stable clusters (Figs. 1 and 2). The robustness of the classification was analyzed by consensus clustering involving *k*-means clustering by resampling (1,000 iterations) randomly selected tumor profiles (Fig. 1). Clinical and pathological characteristics of patients with TNBC are presented according to the four subtypes (Table 1).

**Table 1:** Clinicopathological characteristics of the four TNBC subtypes based on the FUSCC classification criteria

		FUSCC subtypes				
Characteristics	Number	IM	LAR	MES	BLIS	*P*
	(total = 165)	n = 28	n = 29	n = 55	n = 53	
Age, y						0.024
≤50	68	14 (50.0)	6 (20.7)	20 (36.4)	28 (52.8)	
>50	97	14 (50.0)	23 (79.3)	35 (63.6)	25 (47.2)	
Menopause						0.160
Yes	101	16 (57.1)	23 (79.3)	33 (60.0)	29 (54.7)	
No	64	12 (42.9)	6 (20.7)	22 (40.0)	24 (45.3)	
Tunor size, cm						0.409
≤2 cm	58	14 (50.0)	12 (41.4)	15 (27.3)	17 (32.1)	
>2 cm	104	13 (46.4)	17 (58.6)	39 (70.9)	35 (66.0)	
Unknown	3	1 (3.6)	0 (0.0)	1 (1.8)	1 (1.9)	
Tumor grade						0.311
≤ II	32	4 (14.3)	9 (31.0)	13 (23.6)	6 (11.3)	
> II	104	17 (60.7)	17 (58.6)	33 (60.0)	37 (69.8)	
Unknown	29	7 (25.0)	3 (10.3)	9 (16.4)	10 (18.9)	
Ki67, %						0.286
<14	8	0 (0.0)	0 (0.0)	3 (5.5)	5 (9.4)	
≥14	156	28 (100.0)	29 (100.0)	51 (92.7)	48 (90.6)	
Unknown	1	0 (0.0)	0 (0.0)	1 (1.8)	0 (0.0)	
Positive lymph nodes						0.019
0	86	8 (28.6)	13 (44.8)	28 (50.9)	37 (69.8)	
1-3	29	6 (21.4)	5 (17.2)	10 (18.2)	8 (15.1)	
> 3	50	14 (50.0)	11 (37.9)	17 (30.9)	8 (15.1)	
Chemotherapy						0.642
Taxane-based	124	21 (75.0)	22 (75.9)	42 (76.4)	39 (73.6)	
Non-taxane-based	27	5 (17.9)	3 (10.3)	11 (20.0)	8 (15.1)	
Unknown	14	2 (7.1)	4 (13.8)	2 (3.6)	6 (11.3)	
Radiotherapy						0.038
Yes	50	16 (57.1)	9 (31.0)	14 (25.5)	11 (20.8)	
No	103	11 (39.3)	20 (69.0)	37 (67.3)	35 (66.0)	
Unknown	12	1 (3.6)	0 (0.0)	4 (7.3)	6 (11.3)	
Follow up, month						
Median	13.9	14.7	12.4	14.3	12.6	
IQR	8.6–21.1	10.0–22.4	8.6–19.0	10.6–21.5	8.0–18.4	
RFS events	22	5	4	4	9	

*BLIS* basal-like and immune suppressed, *FUSCC* Fudan University Shanghai Cancer Center, *IM* immunomodulatory, *IQR* interquartile range, *LAR* luminal androgen receptor, *MES* mesenchymal-like, *RFS* recurrence-free survival

To understand the nature of each subtype in our system, GO and pathway analyses were performed to determine the top GO and canonical pathways associated with TNBC subtypes. Each subtype, presenting distinct regulator activation and inhibition patterns, was characterized based on the results. The results were correlated with the distribution of the Lehmann/Pietenpol subtypes in our new classification system, which we named the FUSCC classification. Detailed GO and pathway analysis results of each subtype are presented in Additional file 1: Table S1.

### Cluster A: the immunomodulatory (IM) subtype

In concordance with the Lehmann/Pietenpol classification, the IM subtype presented unique GOs and pathways involving immune cell process. These processes included cytokine signaling (cytokine-cytokine receptor interaction), immune cell signaling (T-cell receptor signaling pathway, B-cell receptor signaling pathway), antigen processing and presentation, chemokine signaling pathway, and immune signal transduction pathway (NF-κB signaling pathway). The most upregulated gene functions were tightly connected with immune functions, such as immune response, T cell co-stimulation, and innate immune response. The genes involved in the most significantly upregulated functions are also involved in the immune response process (*CCR2*, *CXCL13*, *CXCL11*, *CD1C*, *CXCL10*, and *CCL5*), which further confirmed the major role of functions related to immunity in this subtype.

### Cluster B: the luminal androgen receptor (LAR) subtype

The LAR type displayed unique GOs, which were highly enriched in hormonally regulated pathways. Androgen and estrogen metabolism, steroid hormone biosynthesis, porphyrin and chlorophyll metabolism, and peroxisome proliferator-activated receptor (PPAR) signaling pathways were significantly elevated in this subtype. Although these tumors were confirmed to be TNBC by immunohistochemical analysis, the gene expression profiling demonstrated an upregulated estrogen signaling pathway. These results suggested this subtype might respond to anti-androgen and traditional anti-estrogen therapies. Thus, to be consistent with previous studies, we classified this as the LAR subtype [18, 30].

### Cluster C: the mesenchymal-like (MES) subtype

**Fig. 3:**
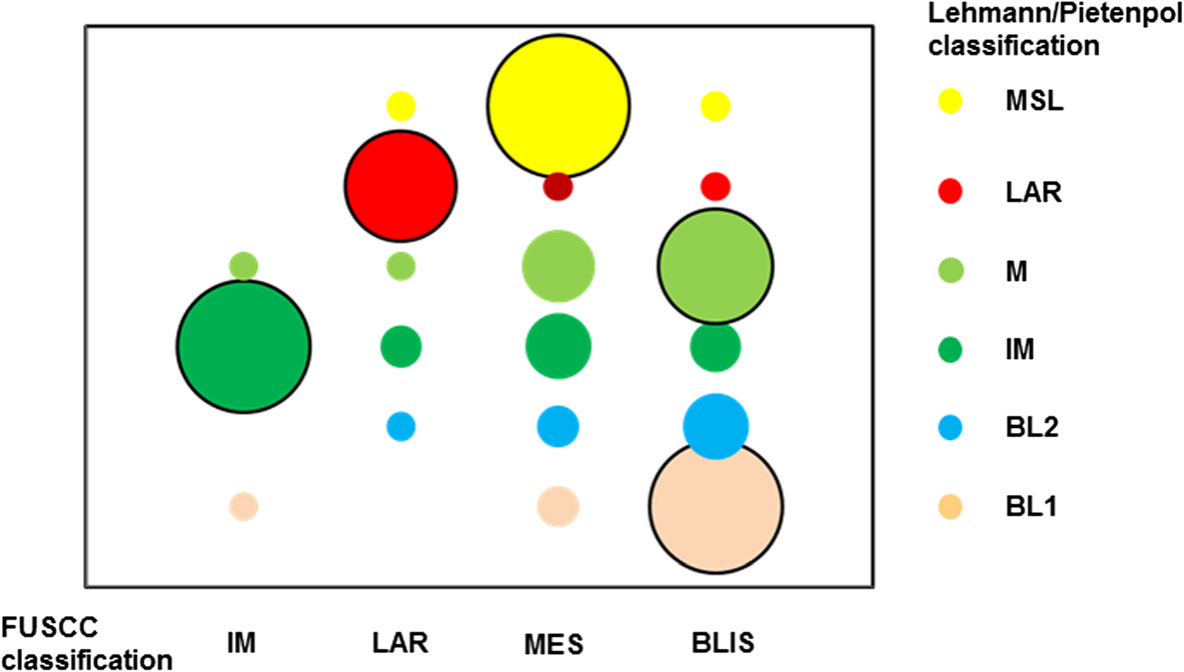
Interaction analysis of the Lehmann/Pietenpol and Fudan University Shanghai Cancer Center (*FUSCC*) classifications. *X-axis* shows the subtypes of the new system. *Circle size* varies in proportion to the number of samples. *MSL* mesenchymal stem-like, *LAR* luminal androgen receptor, *M* claudin-low-enriched mesenchymal, *IM* immunomodulatory, *BL* basal-like, *MES* mesenchymal-like, *BLIS* basal-like and immune-suppressed

This cluster displayed a variety of unique GOs and involved pathways. Enriched pathways in this subtype included extracellular matrix (ECM)-receptor interaction, focal adhesion, and transforming growth factor (TGF)-beta signaling pathway, and processes linked to growth factor signaling pathways (ABC transporter and adipocytokine signaling pathway). Moreover, the MES subtype had low levels of genes related to cell proliferation. The decreased proliferation involved the process of cell division (*CCNE2*, *PARD6B*, *CDCA2*, *KIF2C*, *SKA1*, *NEK2*, *CDK1*, *CDC6*), mitotic cell cycle (*NDC80*, *CENPW*, *MAD2L1*, *CENPI*, *CCNB1*, *CENPF*, *CCNA2*), mitotic prometaphase (*MAD2L1*, *NCAPG*, *SGOL1*, *KIF18A*, *PLK1*), and mitosis (*ASPM*, *HELLS*, *KIF11*, *NUF2*). The major subtype of the Lehmann/Pietenpol classification in this cluster was MSL (Figs. 2 and 3). Altogether, we named this cluster the MES subtype.

### Cluster D: the basal-like and immune-suppressed (BLIS) subtype

For this subtype, the top GOs were enriched in cell division and cell cycle related pathways (mitotic cell cycle, mitotic prometaphase, M phase of mitotic cell cycle, DNA replication, and DNA repair). The enhanced expression of genes associated with proliferation, such as *CENPF*, *BUB1*, *PRC1*, further supported the highly proliferative nature of this subtype. Meanwhile, genes involved in immune responses (immune response and innate immune response), immune cell signaling pathways (T cell co-stimulation, T cell receptor signaling pathway, B cell activation, and dendritic cell chemotaxis) and complement activation processes were significantly downregulated. Previous survival analysis indicated that patients in the BLIS subtype experienced worse RFS compared to other patients. This finding is in concordance with the highly proliferative and immune-suppressed nature of these tumors.

### Association between the FUSCC classification and the Lehmann/Pietenpol classification system

In the Lehmann/Pietenpol classification system [18], TNBCs were classified into seven subtypes (BL1, BL2, LAR, M, IM, MSL, and UNS), whereas according to the FUSCC system, TNBC tumors were divided into four stable subtypes. We then investigated to what extent the FUSCC subtypes based on integrated mRNA-lncRNA expression were associated with the mRNA-based Lehmann/Pietenpol classification (Fig. 2). In Spearman’s correlation analysis, we found that the two classification systems were significantly associated with each other (*P* = 0.039). Further analysis of the distribution of the Lehmann/Pietenpol subtypes in the FUSCC classification system revealed that our subtype IM was nearly identical to the Lehmann/Pietenpol IM type; our subtype LAR mainly contained the LAR type; our subtype MES included all six of the Lehmann/Pietenpol subtypes, with the MSL and M subtypes accounting for the majority; and our subtype BLIS mainly contained the Lehmann/Pietenpol BL1 and M types (Fig. 3).

###  Survival analysis of patients in the four subtypes

**Fig. 4:**
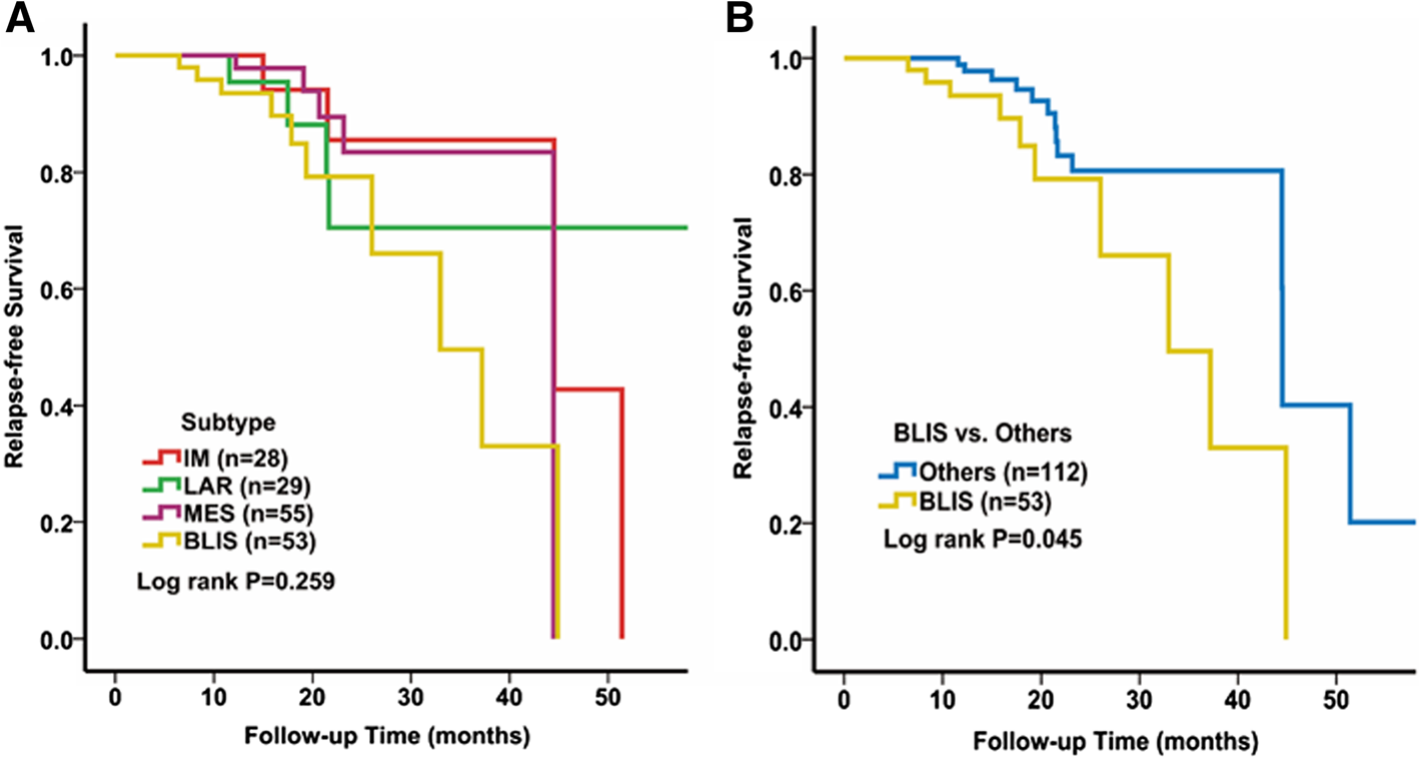
Kaplan-Meier plot and logrank test compared recurrence-free survival (RFS) in different subtypes according to the Fudan University Shanghai Cancer Center (FUSCC) classification. **a** Difference in RFS among four subtypes. **b** RFS in patients with the basal-like 1 (BL1) subtype compared to other subtypes. *IM* immunomodulatory, *LAR* luminal androgen receptor, *MES* mesenchymal-like, *BLIS* basal-like and immune-suppressed

We conducted survival analysis to explore correlations between the four subtypes and RFS. Kaplan–Meier survival analysis showed that there was no significant difference in RFS between the four subtypes (Fig. 4a). However, when we analyzed the data by comparing one subtype with the others, we found that patients in the subtype BLIS experienced worse RFS than the remaining patients with TNBC (Fig. 4b, log rank *P* = 0.045).

### Identifying subtype-specific lncRNAs and their co-expressed mRNAs

**Fig. 5:**
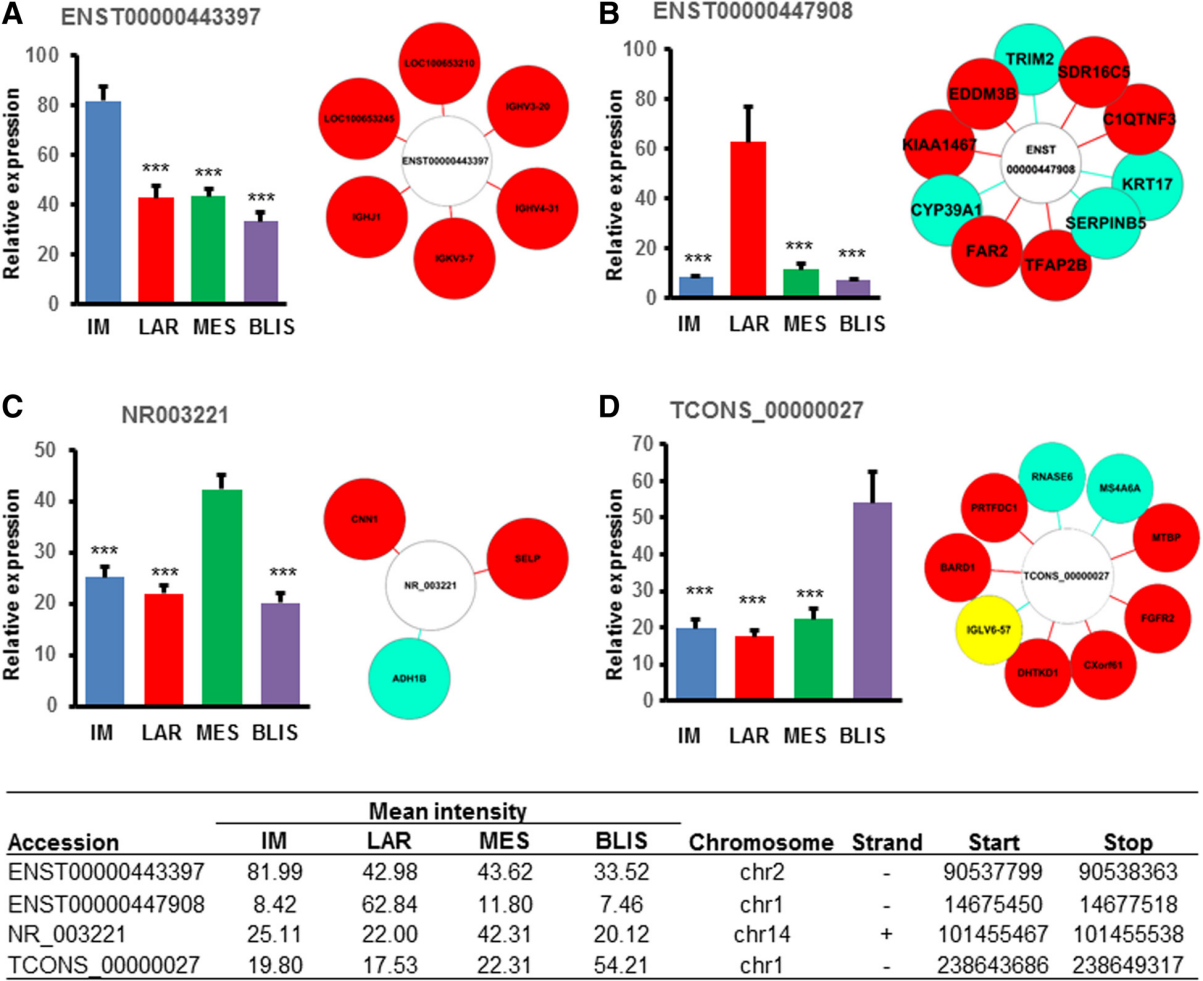
Subtype-specific long noncoding RNAs (lncRNAs) and analysis of their co-expressed messenger RNAs (RNAs). *Table* shows details of the lncRNAs. The highest expression group was selected as the reference. Student’s *t* test, ***P <0.001. *IM* immunomodulatory, *LAR* luminal androgen receptor, *MES* mesenchymal-like, *BLIS* basal-like and immune-suppressed, *MSL* mesenchymal stem-like

We identified differentially expressed lncRNAs in each subtype by comparing the expression intensity of lncRNAs in one specific subtype with the others. Differentially upregulated lncRNAs are as shown in Fig. 5. In the IM subtype, the most upregulated lncRNA was *ENST00000443397*, which was tightly correlated with five mRNAs (Fig. 5a). LncRNA *ENST00000447908* was highly expressed in the LAR subtype, and ten mRNAs were significantly associated with it (Fig. 5b). In the MES subtype, expression of lncRNA *NR_003221* was increased, and it was positively related with two mRNAs and negatively associated with one mRNA (Fig. 5c). LncRNA *TCONS_00000027* was also a novel lncRNA that was highly expressed in the BLIS subtype; nine mRNAs were significantly correlated with it (Fig. 5d). For these four lncRNAs, we further validated their subtype-specific expression in a cohort of breast cancer cell lines and TNBC samples using quantitative real-time PCR (Additional file 2: Figure S5, S6). On further *in situ* hybridization, we validated that lncRNAs *TCONS_00000027* were highly expressed in TNBC samples (Additional file 2: Figure S7). Several other subtype-specific lncRNAs and their basic information are listed in Additional file 2: Figures S1-S4.

## Discussion

In the present study, we established a novel TNBC classification system, the FUSCC classification, by integrating the expression profiles of both mRNAs and lncRNAs. TNBC samples can be clearly classified into four subtypes according to our system: IM, LAR, MES, and BLIS. Each subtype has its own unique transcriptome profile. Furthermore, we filtrated out several subtype-specific lncRNAs and predicted possible functions of these lncRNAs in TNBC biological processes by analyzing the co-expression network between lncRNAs and mRNAs. To the best of our knowledge, the present study is the first to develop a novel TNBC classification system based on the transcriptome profiles of both mRNAs and lncRNAs in a large TNBC cohort.

Several novel findings were revealed in our in-depth transcriptome analysis. First, considering the expanding roles of lncRNAs in tumorigenesis and disease development, we integrated the expression profiles of both mRNAs and lncRNAs in an attempt to comprehensively understand the heterogeneic nature of TNBC. By clustering TNBC samples into four unique subtypes, the FUSCC classification is more simplified than the former Lehmann/Pietenpol system, but we could also recognize some overlaps between the two systems. In the Lehmann/Pietenpol classification, TNBC patients were assigned to six different subtypes according to the combined analysis of 14 publically available RNA profiling datasets [18]. However, subsequent study using the Lehmann/Pietenpol system did not readily distinguish BL1 and BL2 tumors [19]. In our present study, tumors with a basal property were classified into only one subtype (BLIS) that incorporated almost all of the BL1 and BL2 subtypes from the Lehmann/Pietenpol classification. The BLIS subtype is associated with genes involved in proliferation and immunosuppression. Moreover, in the survival analysis, we observed a worse survival outcome for this subtype compared with other subtypes. The results are concordant with those of a previous study in which patients with the same property (basal-like immune-suppressed) had the worst outcome among patients with TNBC [30]. Furthermore, almost all of the BLIS tumors were in the group at high risk of relapse according to the TNBC prognostic signature that we developed (unpublished data). Collectively, these results suggest the aggressive nature of BLIS tumors. Thus, if these results are further validated in other larger populations or prospective cohorts, more aggressive treatment should be tailored for this group of patients.

In the study of Reiche [11], differentially expressed lncRNAs were identified with relation to cancer-related protein-coding genes. This suggests a tight connection between lncRNA and mRNA. Through bioinformatics analyses, we identified several subtype-specific lncRNAs that will be functionally investigated in the future. By analyzing co-expression networks, mRNAs that are highly correlated with the subtype-specific lncRNAs were identified. For example, lncRNA *NR_003221* in the MES subtype is positively correlated with mRNA *CNN1* and *SELP,* but negatively correlated with mRNA *ADH1B. SELP* encodes selectin P, which could mediate the interactions between endothelial cells and leukocytes. A study has shown that high selectin P expression is associated with metastasis of small cell lung cancer [31]. Together with chondroitin sulfate proteoglycan 4, selectin P can bind to highly metastatic breast cancer cells and removal of selectin P ligand could reduce metastatic lung colonization [32]. *ADH1B* encodes alcohol dehydrogenase. Decreased expression of *ADH1B* gene has been proved to be associated with disease progression in human colorectal cancer [33]. Taken together, we hypothesize that lncRNA *NR_003221* may play a role in cancer development by promoting cell metastasis.

Our study has several limitations. First, the new classification has not yet been validated in other cohorts. Due to the limited data on lncRNA expression in TNBC, we did not validate the system in publicly available datasets. Second, even though GO and pathway analyses were performed, the nature of each subtype was not thoroughly clarified, and in particular, lacked support from functional experiments. Third, the follow-up time of the prospective observational study was relatively short, and may have resulted in the marginal difference in survival (BLIS vs. others). Further updating the follow-up data might help clarify the association between subtypes and survival outcome. Last, compared with other available technology, such as RNAseq, the HTA2.0 cannot identify novel lncRNAs. Therefore, our future work will focus on updating the follow up of the cohort, recruiting independent cohorts to validate the FUSCC classifier and investigating the functions of novel lncRNAs in each subtype.

## Conclusions

We have developed a novel TNBC subtyping system, assigning TNBC patients to four distinct subtypes by integrating both mRNA and lncRNA expression profiles. In addition, we revealed a number of novel subtype-specific lncRNAs that help elucidate the nature of each subtype. Once further validated in a larger population, the subtype system could facilitate individualized treatment of TNBC.

## Additional files

Additional file 1: Top gene ontologies and pathways involved in each subtype. Additional file 2: Figure S1. Subtype-specific long noncoding RNA (lncRNAs) in the immunomodulatory subgroup. Figure S2 Subtype-specific lncRNA in the luminal androgen receptor subgroup. Figure S3 Subtype-specific lncRNAs in the mesenchymal-like subgroup. Figure S4 Subtype-specific lncRNAs in the basal-like and immune-suppressed subgroup. Figure S5 Validation of subtype-specific lncRNAs using quantitative real-time PCR in triple-negative breast cancer. Figure S6 Validation of subtype-specific lncRNAs using quantitative real time PCR in breast cancer cell lines. Figure S7 Validation of lncRNAs TCONS_00000027 in breast cancer tissue microarray using RNA *in situ* hybridization technology. (PPTX 3172 kb)

## Abbreviations

BL1: basal-like-1
BL2: basal-like-2
BLIS: basal-like and immune-suppressed
CDF: empirical cumulative distribution function
ECM: extracellular matrix
ER: estrogen receptor
FUSCC: Fudan University Shanghai Cancer Center
GO: Gene Ontology
HER2: human epidermal growth factor receptor 2
HTA 2.0: Human Transcriptome Array 2.0
KEGG: Kyoto Encyclopedia of Genes and Genomes database
IM: immunomodulatory
LAR: luminal androgen receptor
lncRNA: long noncoding RNA
M: claudin-low-enriched mesenchymal
MES: mesenchymal-like
MSL: mesenchymal stem-like
mRNA: messenger RNA
NCBI: National Center for Biotechnology Information
PR: progesterone receptor
RFS: recurrence-free survival
TGF: transforming growth factor
TNBC: triple-negative breast cancer
UNS: unstable
