# Supplementary material for: Comprehensive transcriptome analysis identifies novel molecular subtypes and subtype-specific RNAs of triple-negative breast cancer
Source: Breast Cancer Res. 2016 Mar 15;18:33. doi: 10.1186/s13058-016-0690-8 (PMC4791797; doi:10.1186/s13058-016-0690-8)
Supplement: Additional file 1: — Top gene ontologies and pathways involved in each subtype. [file 13058_2016_690_MOESM1_ESM.docx]

**Table S1**. Top gene ontologies and pathways involved in each subtype.

| Subtypes | Top gene ontologies | | Top pathways |
| --- | --- | --- | --- |
| Immuno-  modulatory  (IM) | | Immune response  T cell costimulation  Innate immune response  Defense response to virus  Inflammatory response  Response to virus  T cell receptor signaling pathway  B cell activation  Cytokine-mediated signaling pathway  Interferon-gamma-mediated signaling pathway  Regulation of immune response  Dendritic cell chemotaxis  Cell surface receptor signaling pathway  Complement activation, classical pathway  Positive regulation of T cell proliferation  B cell receptor signaling pathway | Cell adhesion molecules (CAMs)  Intestinal immune network for IgA production  Influenza A  Hematopoietic cell lineage  Rheumatoid arthritis  Chemokine signaling pathway  Cytokine-cytokine receptor interaction  Leishmaniasis  Autoimmune thyroid disease  Allograft rejection  Staphylococcus aureus infection  Graft-versus-host disease  Type I diabetes mellitus  Herpes simplex infection  Measles  Antigen processing and presentation  NF-kappa B signaling pathway |
| Luminal androgen receptor  (LAR) | | Small molecule metabolic process  Xenobiotic metabolic process  Transmembrane transport  Fatty acid biosynthetic process  Androgen biosynthetic process  Androgen and estrogen metabolism  Cellular nitrogen compound metabolic process  Steroid metabolic process  Transport  Lipid metabolic process  Cellular lipid metabolic process  Activation of prostate induction by Androgen receptor signaling pathway | Metabolic pathways  Steroid hormone biosynthesis  Metabolism of xenobiotics by cytochrome P450  Drug metabolism - cytochrome P450  Chemical carcinogenesis  Pentose and glucuronate interconversions  Starch and sucrose metabolism  Porphyrin and chlorophyll metabolism  PPAR signaling pathway  Drug metabolism - other enzymes  Peroxisome |
| Mesenchymal-  like  (MES) | | *Cell division*  *Mitotic cell cycle*  *Mitotic prometaphase*  *M phase of mitotic cell cycle*  *Mitosis*  *Extracellular matrix organization*  *Apoptotic process*  *Negative regulation of apoptotic process*  *Chromosome segregation*  *Nucleosome assembly*  *Regulation of cyclin-dependent protein Serine/threonine kinase activity*  *Collagen catabolic process*  *Cell adhesion*  *Cell proliferation* | Cytokine-cytokine receptor interaction  Staphylococcus aureus infection  PPAR signaling pathway  Proteoglycans in cancer  Focal adhesion  ABC transporters  Chemokine signaling pathway  Adipocytokine signaling pathway  PI3K-Akt signaling pathway  Drug metabolism - cytochrome P450  Tyrosine metabolism  TGF-beta signaling pathway  Pathways in cancer  Metabolic pathways |
| Basal-like and immune suppressed  (BLIS) | | Cell division  Mitotic cell cycle  Mitotic prometaphase  M phase of mitotic cell cycle  Mitosis  Cell adhesion  Serine/threonine kinase activity  Epidermis development  Cell proliferation  DNA replication  DNA repair  Mitotic anaphase  Apoptotic process  Positive regulation of apoptotic process  *Immune response*  *Innate immune response*  *Cell adhesion*  *Small molecule metabolic process*  *Blood coagulation*  *Inflammatory response*  *T cell costimulation*  *Complement activation, classical pathway*  *Platelet degranulation* | Systemic lupus erythematosus  Alcoholism  Cell cycle  Pathways in cancer  ECM-receptor interaction  Oocyte meiosis  Cell adhesion molecules (CAMs)  Progesterone-mediated oocyte maturation  p53 signaling pathway  Viral carcinogenesis  Transcriptional misregulation in cancer  Arrhythmogenic right ventricular Cardiomyopathy (ARVC)  Focal adhesion  Wnt signaling pathway  Proteoglycans in cancer  Small cell lung cancer  PI3K-Akt signaling pathway |

Notes: Gene ontology names in italics present downregulated function in the subtype. Others are upregulated.
