# Supplementary material for: Comprehensive transcriptome analysis identifies novel molecular subtypes and subtype-specific RNAs of triple-negative breast cancer
Source: Breast Cancer Res. 2016 Mar 15;18:33. doi: 10.1186/s13058-016-0690-8 (PMC4791797; doi:10.1186/s13058-016-0690-8)
Supplement: Additional file 2: Figure S1. — Subtype-specific long noncoding RNA (lncRNAs) in the immunomodulatory subgroup. Figure S2 Subtype-specific lncRNA in the luminal androgen receptor subgroup. Figure S3 Subtype-specific lncRNAs in the mesenchymal-like subgroup. Figure S4 Subtype-specific lncRNAs in the basal-like and immune-suppressed subgroup. Figure S5 Validation of subtype-specific lncRNAs using quantitative real-time PCR in triple-negative breast cancer. Figure S6 Validation of subtype-specific lncRNAs using quantitative real time PCR in breast cancer cell lines. Figure S7 Validation of lncRNAs TCONS_00000027 in breast cancer tissue microarray using RNA in situ hybridization technology. (PPTX 3172 kb) [file 13058_2016_690_MOESM2_ESM.pptx]

## Slide 1
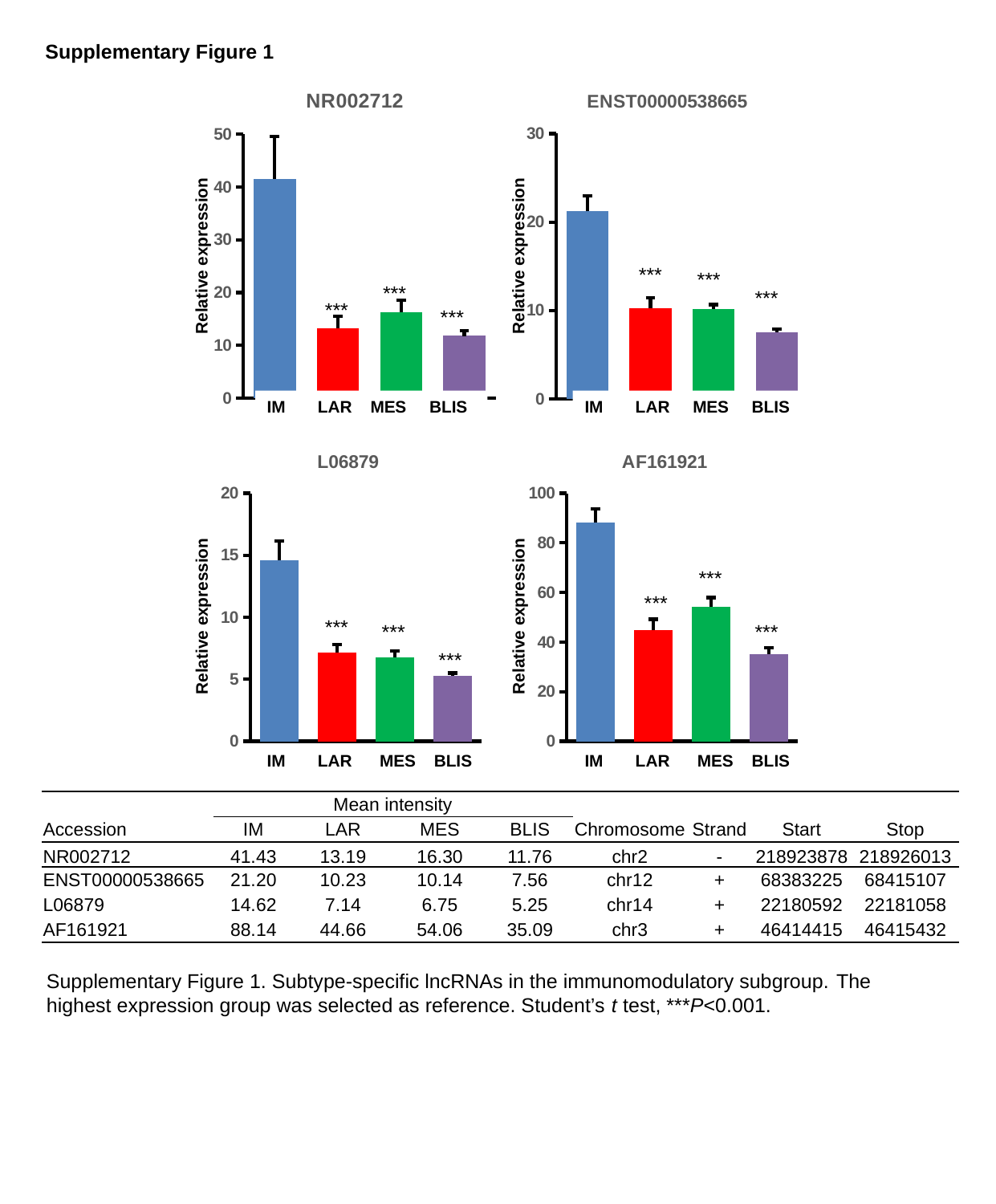

Supplementary Figure 1
### Chart: NR002712
| Category | TC02004815.hg.1 |
|---|---|
| A | 41.4344 |
| B | 13.1948 |
| C | 16.3035 |
| D | 11.7554 |
### Chart: ENST00000538665
| Category | TC12000599.hg.1 |
|---|---|
| A | 21.1964 |
| B | 10.23 |
| C | 10.1448 |
| D | 7.5597 |Relative expression
Relative expression
IM LAR MES BLIS
IM LAR MES BLIS
### Chart: L06879
| Category | TC14001586.hg.1 |
|---|---|
| A | 14.6172 |
| B | 7.1445 |
| C | 6.7458 |
| D | 5.2502 |
### Chart: AF161921
| Category | TC03002321.hg.1 |
|---|---|
| A | 88.1355 |
| B | 44.6639 |
| C | 54.0591 |
| D | 35.088 |Relative expression
Relative expression
IM LAR MES BLIS
IM LAR MES BLIS
***
***
***
***
***
***
***
***
***
***
***
***
| | Mean intensity | | | | | | | |
| --- | --- | --- | --- | --- | --- | --- | --- | --- |
| Accession | IM | LAR | MES | BLIS | Chromosome | Strand | Start | Stop |
| NR002712 | 41.43 | 13.19 | 16.30 | 11.76 | chr2 | - | 218923878 | 218926013 |
| ENST00000538665 | 21.20 | 10.23 | 10.14 | 7.56 | chr12 | + | 68383225 | 68415107 |
| L06879 | 14.62 | 7.14 | 6.75 | 5.25 | chr14 | + | 22180592 | 22181058 |
| AF161921 | 88.14 | 44.66 | 54.06 | 35.09 | chr3 | + | 46414415 | 46415432 |
Supplementary Figure 1. Subtype-specific lncRNAs in the immunomodulatory subgroup. The highest expression group was selected as reference. Student’s t test, ***P<0.001.

## Slide 2
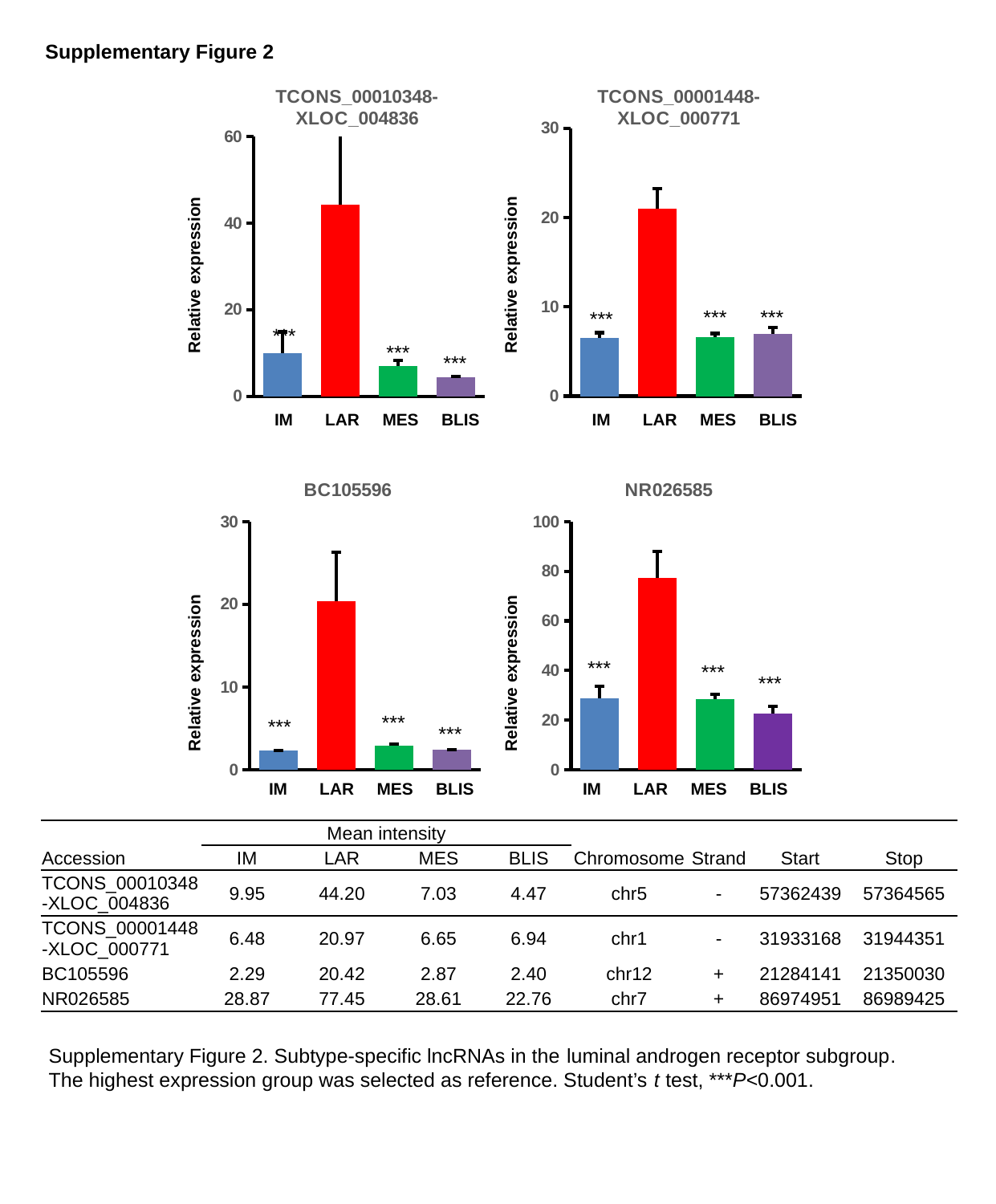

Supplementary Figure 2
### Chart: TCONS_00010348-XLOC_004836
| Category | TC05003016.hg.1 |
|---|---|
| A | 9.9524 |
| B | 44.1954 |
| C | 7.0279 |
| D | 4.4652 |Relative expression
IM LAR MES BLIS
### Chart: TCONS_00001448-XLOC_000771
| Category | TC01005380.hg.1 |
|---|---|
| A | 6.4807 |
| B | 20.968 |
| C | 6.6475 |
| D | 6.9426 |Relative expression
IM LAR MES BLIS
***
***
***
***
***
***
### Chart: BC105596
| Category | TC12002283.hg.1 |
|---|---|
| A | 2.2868 |
| B | 20.4215 |
| C | 2.868 |
| D | 2.3993 |Relative expression
IM LAR MES BLIS
### Chart: NR026585
| Category | TC07002442.hg.1 |
|---|---|
| A | 28.8666 |
| B | 77.445 |
| C | 28.612 |
| D | 22.764 |Relative expression
IM LAR MES BLIS
***
***
***
***
***
***
| | Mean intensity | | | | | | | |
| --- | --- | --- | --- | --- | --- | --- | --- | --- |
| Accession | IM | LAR | MES | BLIS | Chromosome | Strand | Start | Stop |
| TCONS\_00010348-XLOC\_004836 | 9.95 | 44.20 | 7.03 | 4.47 | chr5 | - | 57362439 | 57364565 |
| TCONS\_00001448-XLOC\_000771 | 6.48 | 20.97 | 6.65 | 6.94 | chr1 | - | 31933168 | 31944351 |
| BC105596 | 2.29 | 20.42 | 2.87 | 2.40 | chr12 | + | 21284141 | 21350030 |
| NR026585 | 28.87 | 77.45 | 28.61 | 22.76 | chr7 | + | 86974951 | 86989425 |
Supplementary Figure 2. Subtype-specific lncRNAs in the luminal androgen receptor subgroup. The highest expression group was selected as reference. Student’s t test, ***P<0.001.

## Slide 3
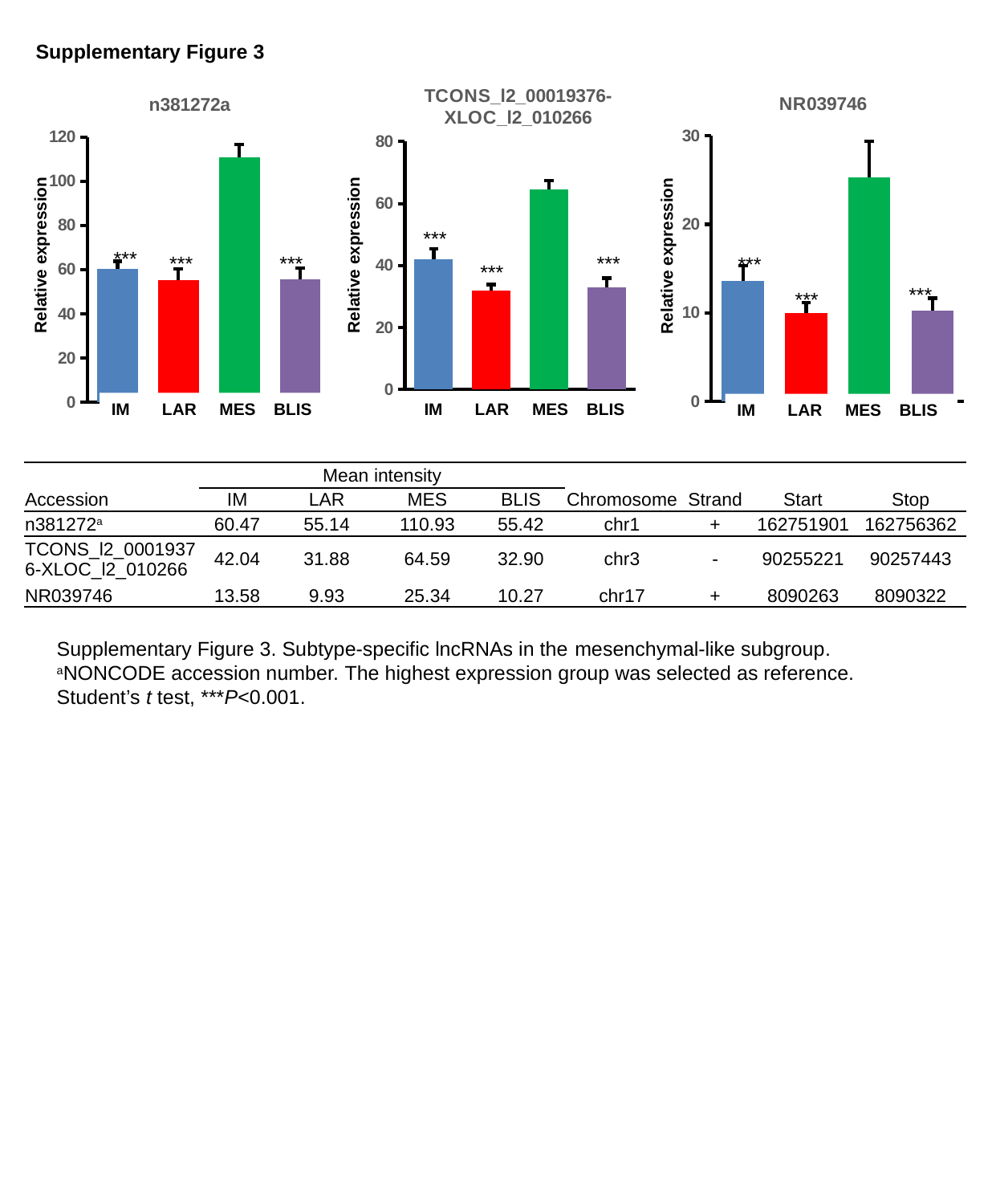

Supplementary Figure 3
### Chart: NR039746
| Category | TC17000132.hg.1 |
|---|---|
| A | 13.5815 |
| B | 9.934 |
| C | 25.3373 |
| D | 10.2734 |
### Chart: n381272a
| Category | TC01004802.hg.1 |
|---|---|
| A | 60.465 |
| B | 55.1442 |
| C | 110.9282 |
| D | 55.421 |Relative expression
IM LAR MES BLIS
### Chart: TCONS_l2_00019376-XLOC_l2_010266
| Category | TC03002992.hg.1 |
|---|---|
| A | 42.0388 |
| B | 31.8823 |
| C | 64.589 |
| D | 32.9031 |Relative expression
IM LAR MES BLIS
***
Relative expression
***
***
***
***
***
***
***
***
IM LAR MES BLIS
| | Mean intensity | | | | | | | |
| --- | --- | --- | --- | --- | --- | --- | --- | --- |
| Accession | IM | LAR | MES | BLIS | Chromosome | Strand | Start | Stop |
| n381272a | 60.47 | 55.14 | 110.93 | 55.42 | chr1 | + | 162751901 | 162756362 |
| TCONS\_l2\_00019376-XLOC\_l2\_010266 | 42.04 | 31.88 | 64.59 | 32.90 | chr3 | - | 90255221 | 90257443 |
| NR039746 | 13.58 | 9.93 | 25.34 | 10.27 | chr17 | + | 8090263 | 8090322 |
Supplementary Figure 3. Subtype-specific lncRNAs in the mesenchymal-like subgroup. aNONCODE accession number. The highest expression group was selected as reference. Student’s t test, ***P<0.001.

## Slide 4
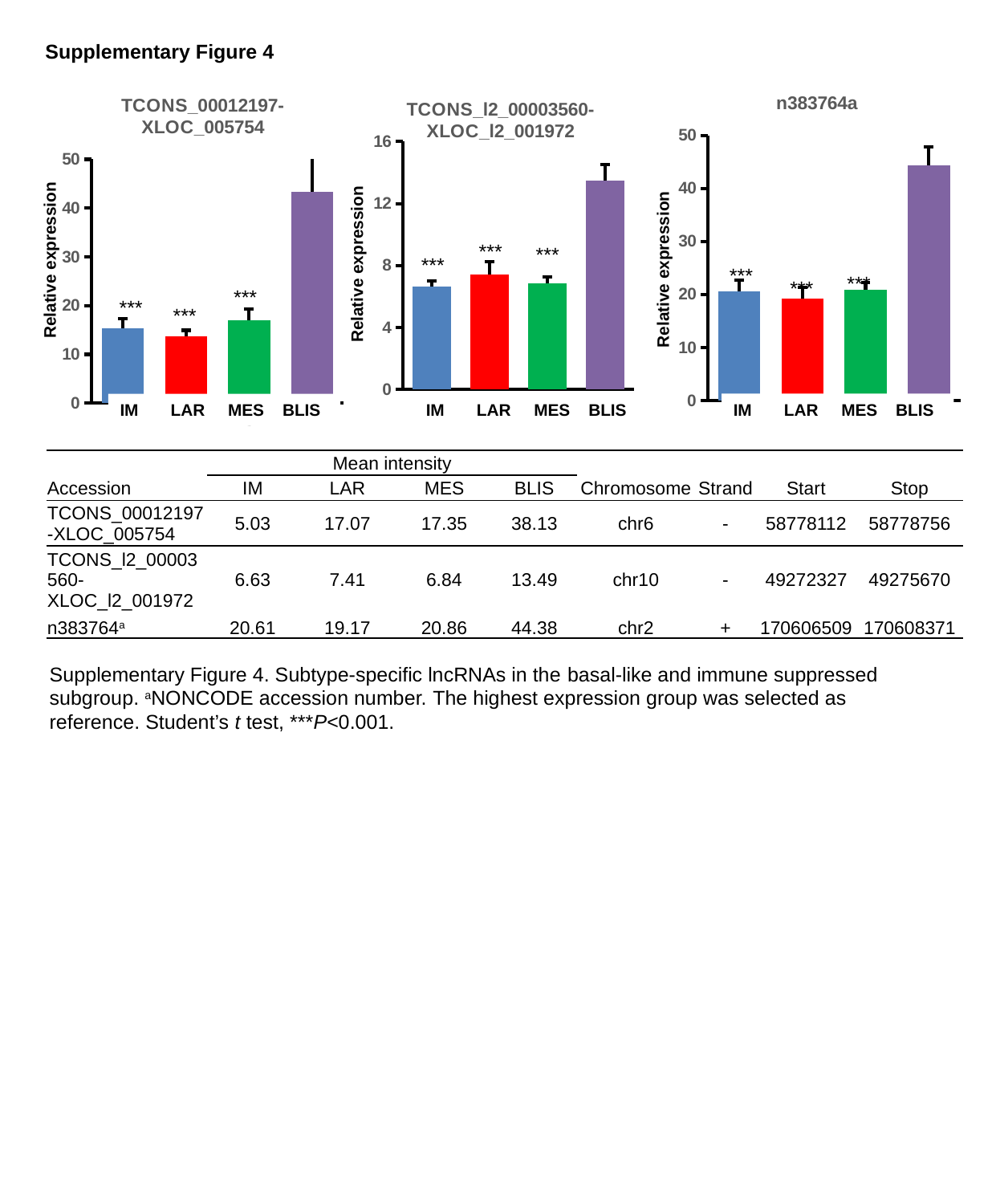

Supplementary Figure 4
### Chart: n383764a
| Category | TC02003679.hg.1 |
|---|---|
| A | 20.6132 |
| B | 19.1744 |
| C | 20.8647 |
| D | 44.3815 |
### Chart: TCONS_00012197-XLOC_005754
| Category | TC01004013.hg.1 |
|---|---|
| A | 15.3387 |
| B | 13.6563 |
| C | 16.9943 |
| D | 43.3948 |Relative expression
IM LAR MES BLIS
### Chart: TCONS_l2_00003560-XLOC_l2_001972
| Category | TC10002627.hg.1 |
|---|---|
| A | 6.6328 |
| B | 7.4129 |
| C | 6.8421 |
| D | 13.4887 |Relative expression
IM LAR MES BLIS
Relative expression
IM LAR MES BLIS
***
***
***
***
***
***
***
***
***
| | Mean intensity | | | | | | | |
| --- | --- | --- | --- | --- | --- | --- | --- | --- |
| Accession | IM | LAR | MES | BLIS | Chromosome | Strand | Start | Stop |
| TCONS\_00012197-XLOC\_005754 | 5.03 | 17.07 | 17.35 | 38.13 | chr6 | - | 58778112 | 58778756 |
| TCONS\_l2\_00003560-XLOC\_l2\_001972 | 6.63 | 7.41 | 6.84 | 13.49 | chr10 | - | 49272327 | 49275670 |
| n383764a | 20.61 | 19.17 | 20.86 | 44.38 | chr2 | + | 170606509 | 170608371 |
Supplementary Figure 4. Subtype-specific lncRNAs in the basal-like and immune suppressed subgroup. aNONCODE accession number. The highest expression group was selected as reference. Student’s t test, ***P<0.001.

## Slide 5
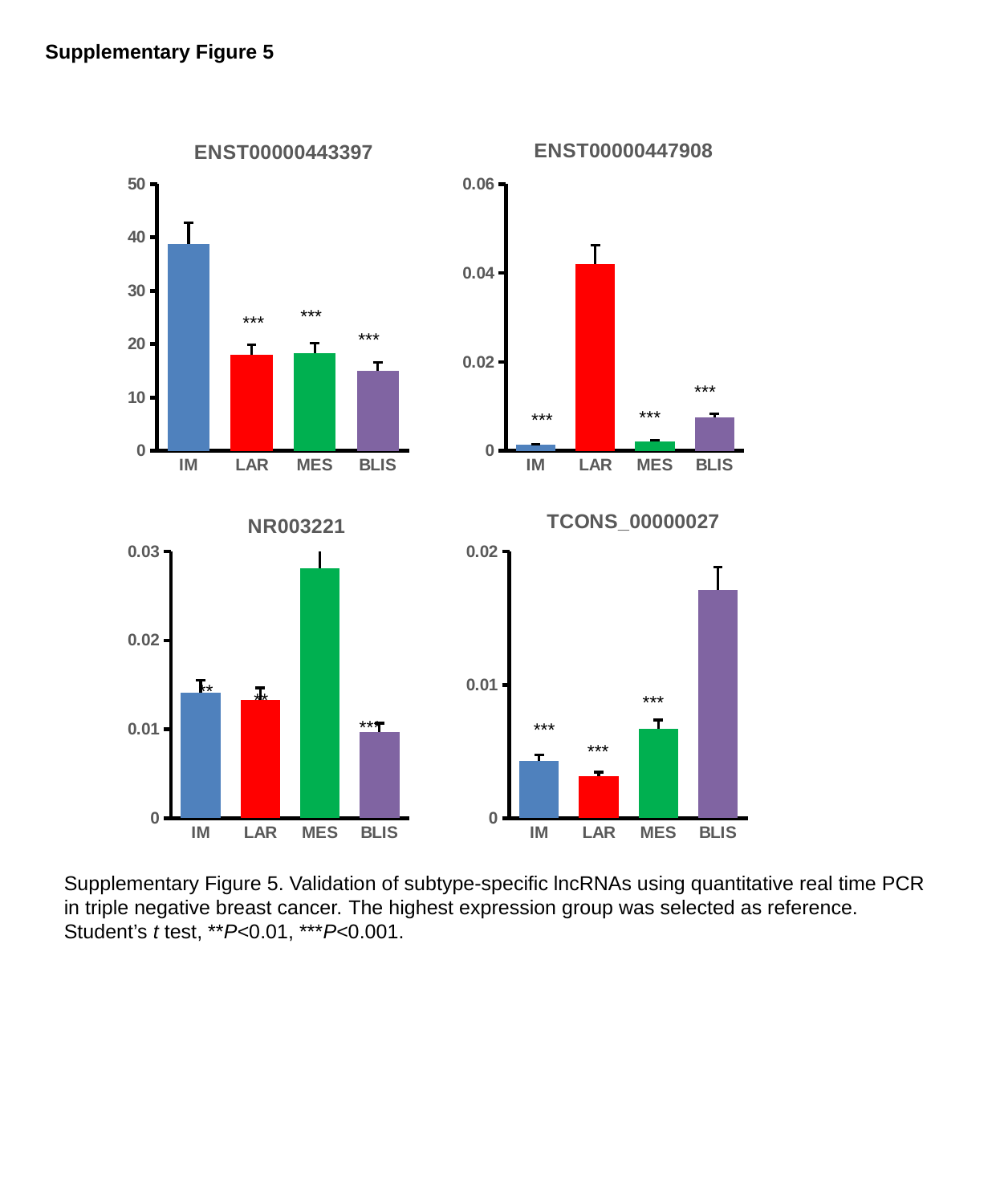

Supplementary Figure 5
### Chart: ENST00000443397
| Category | ENST00000443397 |
|---|---|
| IM | 38.840434350566724 |
| LAR | 18.04281531495809 |
| MES | 18.360790209139047 |
| BLIS | 15.050367819356753 |
### Chart: ENST00000447908
| Category | ENST00000447908 |
|---|---|
| IM | 0.001352421012851287 |
| LAR | 0.042029682431702214 |
| MES | 0.002118781945159491 |
| BLIS | 0.007541167255397599 |***
***
***
***
***
***
### Chart: NR003221
| Category | NR003221 |
|---|---|
| IM | 0.01410934791167048 |
| LAR | 0.0133314371532402 |
| MES | 0.028135922070745906 |
| BLIS | 0.009721675488283368 |
### Chart: TCONS_00000027
| Category | TCONS_00000027 |
|---|---|
| IM | 0.004303089665324191 |
| LAR | 0.0031426059844323383 |
| MES | 0.006695460736131821 |
| BLIS | 0.017119207800760466 |**
**
***
***
***
***
Supplementary Figure 5. Validation of subtype-specific lncRNAs using quantitative real time PCR in triple negative breast cancer. The highest expression group was selected as reference. Student’s t test, **P<0.01, ***P<0.001.

## Slide 6
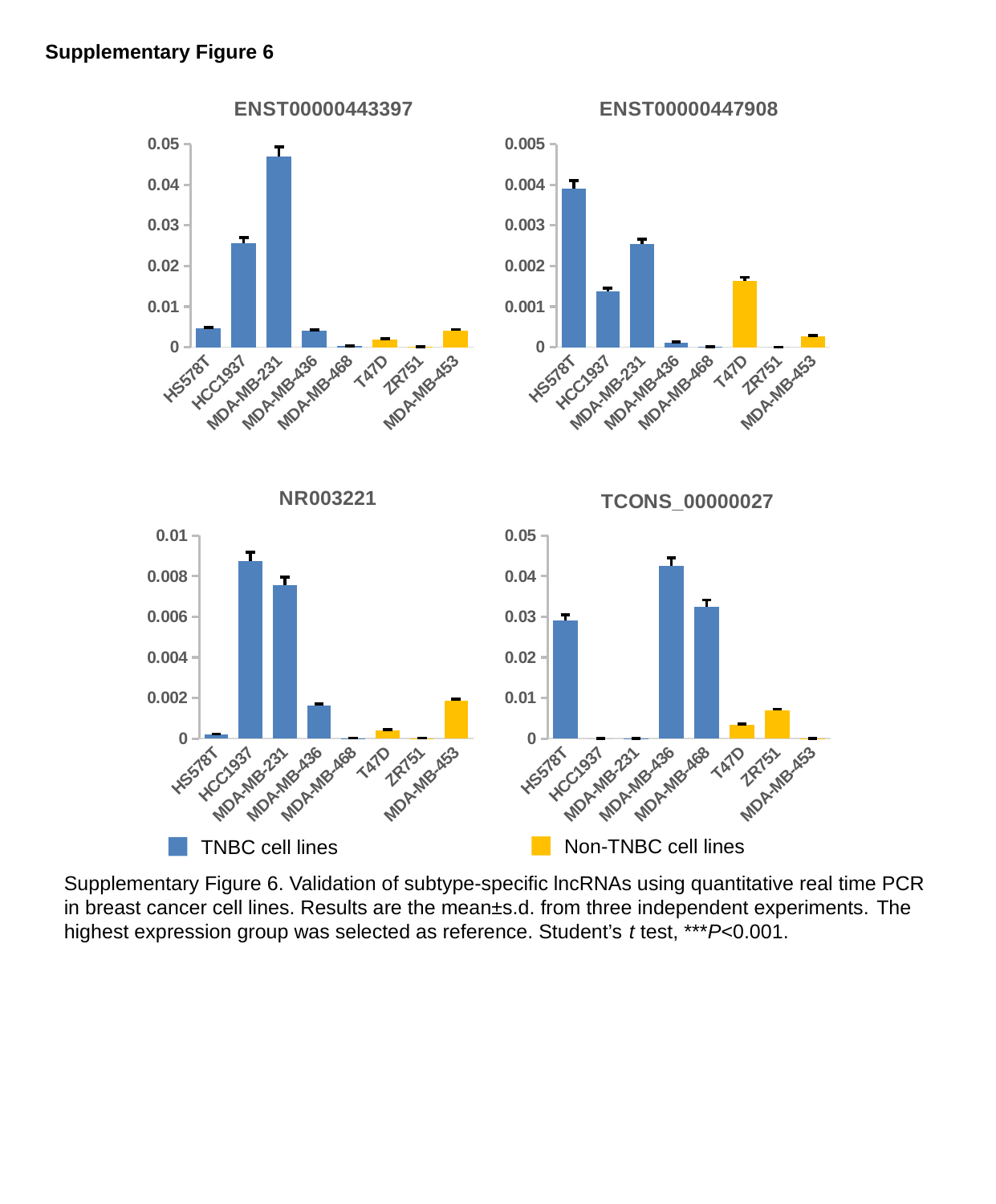

Supplementary Figure 6
### Chart:
| Category | ENST00000443397 |
|---|---|
| HS578T | 0.004576299957810437 |
| HCC1937 | 0.025649493841281904 |
| MDA-MB-231 | 0.04693669273082612 |
| MDA-MB-436 | 0.004014728102908527 |
| MDA-MB-468 | 0.0002653437154494781 |
| T47D | 0.001979016011373367 |
| ZR751 | 7.262885357739014e-05 |
| MDA-MB-453 | 0.004150168612660199 |
### Chart:
| Category | ENST00000447908 |
|---|---|
| HS578T | 0.003900702447784328 |
| HCC1937 | 0.0013822960340612305 |
| MDA-MB-231 | 0.002532005639379826 |
| MDA-MB-436 | 0.00011709271979959949 |
| MDA-MB-468 | 9.033102437171689e-06 |
| T47D | 0.0016396659813261404 |
| ZR751 | 0.0 |
| MDA-MB-453 | 0.0002753397137804575 |
### Chart:
| Category | NR003221 |
|---|---|
| HS578T | 0.00021209829834175264 |
| HCC1937 | 0.0087507719175658 |
| MDA-MB-231 | 0.00756490492723271 |
| MDA-MB-436 | 0.0016392648353667641 |
| MDA-MB-468 | 2.1750717227218446e-05 |
| T47D | 0.00042356392290461407 |
| ZR751 | 2.4405023502982988e-05 |
| MDA-MB-453 | 0.0018527851790924605 |
### Chart:
| Category | TCONS_00000027 |
|---|---|
| HS578T | 0.029055699552443016 |
| HCC1937 | 0.0 |
| MDA-MB-231 | 9.988986007742486e-06 |
| MDA-MB-436 | 0.042440800160018874 |
| MDA-MB-468 | 0.03254114142598223 |
| T47D | 0.0034521407444226297 |
| ZR751 | 0.006907937123058103 |
| MDA-MB-453 | 3.365632857391365e-05 |Non-TNBC cell lines
TNBC cell lines
Supplementary Figure 6. Validation of subtype-specific lncRNAs using quantitative real time PCR in breast cancer cell lines. Results are the mean±s.d. from three independent experiments. The highest expression group was selected as reference. Student’s t test, ***P<0.001.

## Slide 7
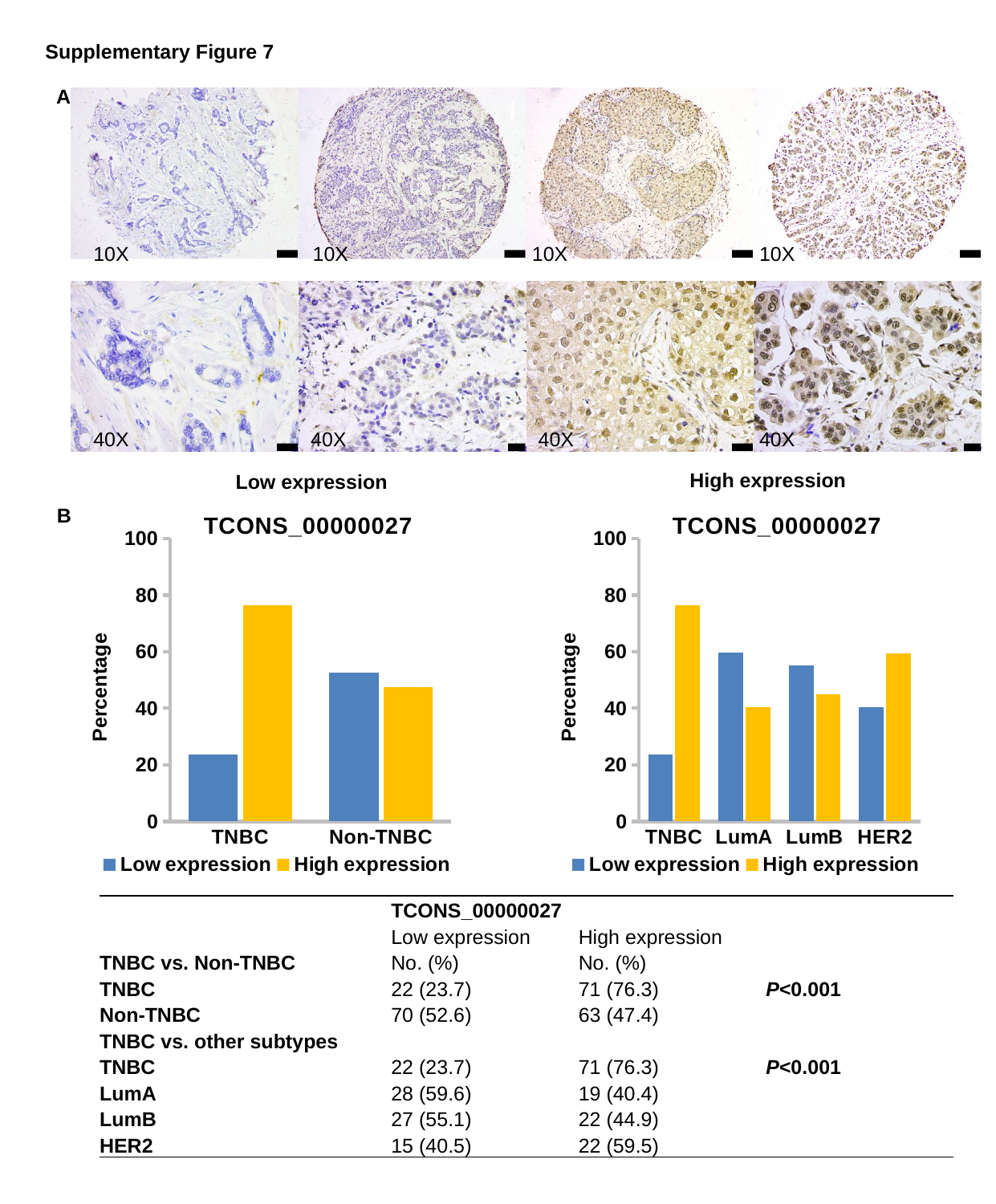

Supplementary Figure 7
A
10X
10X
10X
10X
40X
40X
40X
40X
High expression
Low expression
### Chart: TCONS_00000027
| Category | Low expression | High expression |
|---|---|---|
| TNBC | 23.7 | 76.3 |
| Non-TNBC | 52.6 | 47.4 |
### Chart: TCONS_00000027
| Category | Low expression | High expression |
|---|---|---|
| TNBC | 23.7 | 76.3 |
| LumA | 59.599999999999994 | 40.400000000000006 |
| LumB | 55.1 | 44.9 |
| HER2 | 40.5 | 59.5 |B
| | TCONS\_00000027 | | |
| --- | --- | --- | --- |
| | Low expression | High expression | |
| TNBC vs. Non-TNBC | No. (%) | No. (%) | |
| TNBC | 22 (23.7) | 71 (76.3) | P<0.001 |
| Non-TNBC | 70 (52.6) | 63 (47.4) | |
| TNBC vs. other subtypes | | | |
| TNBC | 22 (23.7) | 71 (76.3) | P<0.001 |
| LumA | 28 (59.6) | 19 (40.4) | |
| LumB | 27 (55.1) | 22 (44.9) | |
| HER2 | 15 (40.5) | 22 (59.5) | |

## Slide 8
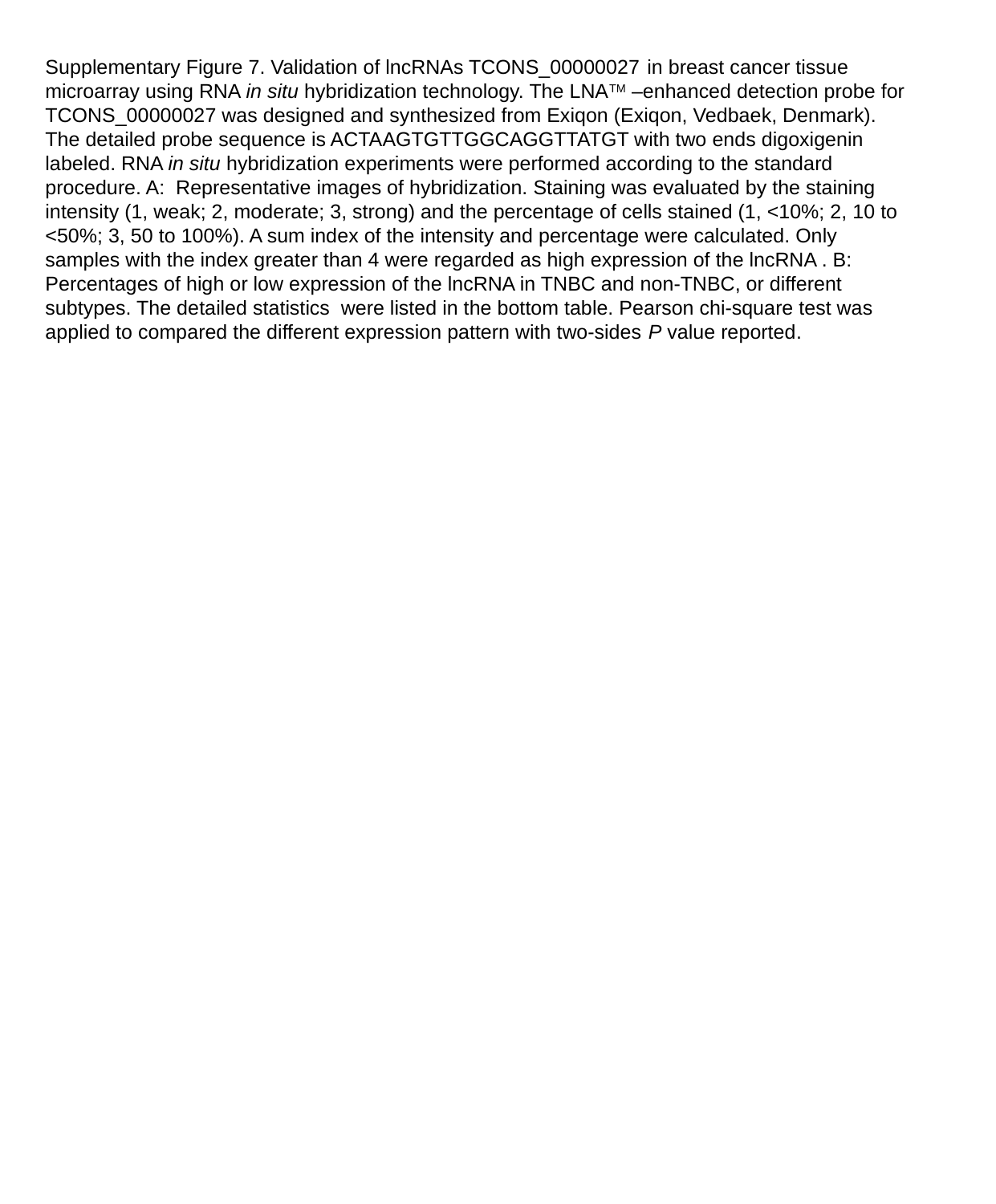

# Supplementary Figure 7. Validation of lncRNAs TCONS_00000027	in breast cancer tissue microarray using RNA in situ hybridization technology. The LNATM –enhanced detection probe for TCONS_00000027 was designed and synthesized from Exiqon (Exiqon, Vedbaek, Denmark). The detailed probe sequence is ACTAAGTGTTGGCAGGTTATGT with two ends digoxigenin labeled. RNA in situ hybridization experiments were performed according to the standard procedure. A: Representative images of hybridization. Staining was evaluated by the staining intensity (1, weak; 2, moderate; 3, strong) and the percentage of cells stained (1, <10%; 2, 10 to <50%; 3, 50 to 100%). A sum index of the intensity and percentage were calculated. Only samples with the index greater than 4 were regarded as high expression of the lncRNA . B: Percentages of high or low expression of the lncRNA in TNBC and non-TNBC, or different subtypes. The detailed statistics were listed in the bottom table. Pearson chi-square test was applied to compared the different expression pattern with two-sides P value reported.
